# Effects of urea topdressing time on yield, nitrogen utilization, and quality of mechanical direct-seeding hybrid *indica* rice under slow-mixed fertilizer base application

**DOI:** 10.3389/fpls.2024.1400146

**Published:** 2024-05-10

**Authors:** Yongjian Sun, Mengwen Xing, Ziting He, Yuanyuan Sun, Yuqian Deng, Yongheng Luo, Xuefang Chen, Yun Cao, Wenbo Xiong, Xinghai Huang, Pengxin Deng, Min Luo, Zhiyuan Yang, Zongkui Chen, Jun Ma

**Affiliations:** ^1^State Key Laboratory of Crop Gene Exploration and Utilization in Southwest China, Sichuan Agricultural University, Chengdu, China; ^2^Ecophysiology and Cultivation Key Laboratory of Sichuan Province, Sichuan Agricultural University, Chengdu, China; ^3^Sichuan Agricultural Meteorological Center, Sichuan Meteorological Bureau, Chengdu, China; ^4^Rongxian Agricultural Technology Extension Center, Rongxian Agricultural and Rural Bureau, Rongxian, China

**Keywords:** slow-mixed fertilizer, urea-N topdressing, direct-seeding rice, yield, rice quality

## Abstract

**Introduction:**

The use of controlled-release nitrogen (N) fertilizers has been shown to improve yield and N-use efficiency (NUE) in mechanical transplanted rice. However, the fertilizer requirements for mechanical direct-seeding rice differ from those for mechanical transplanted rice. The effects of controlled-release fertilizers on yield, NUE, and quality in mechanical direct-seeding rice are still unknown.

**Methods:**

Hybrid *indica* rice varieties Yixiangyou 2115 and Fyou 498 were used as test materials, and slow-mixed N fertilizer (120 kg hm^-2^) as a base (N_1_), N_1_+urea-N (30 kg hm^-2^) once as a base (N_2_), N_1_+urea-N (30 kg hm^-2^) topdressing at the tillering stage (N_3_), N_1_+urea-N (30 kg hm^-2^) topdressing at the booting stage (N_4_) four N fertilizer management to study their impact on the yield, NUE and quality of mechanical direct-seeding rice.

**Results and discussion:**

Compared with Yixiangyou 2115, Fyou 498 significantly increased photosynthetic potential, population growth rate, root vigor, and N transport rate by 3.34–23.88%. This increase further resulted in a significant improvement in the yield and NUE of urea-N topdressing by 1.73–5.95 kg kg^-1^. However, Fyou 498 showed a significant decrease in the head rice rate and taste value by 3.34–7.67%. All varieties were treated with N_4_ that significantly increase photosynthetic potential and population growth rate by 15.41–62.72%, reduce the decay rate of root vigor by 5.01–21.39%, promote the N transport amount in stem-sheaths (leaves) by 13.54–59.96%, and then significantly increase the yields by 4.45–20.98% and NUE of urea-N topdressing by 5.20–45.56 kg kg^-1^. Moreover, the rice processing and taste values were optimized using this model. Correlation analysis revealed to achieve synergistic enhancement of high-yield, high-quality, and high-NUE in rice, it is crucial to focus on increasing photosynthetic potential, population growth rate, and promoting leaf N transport. Specifically, increasing the contribution rate of N transport in stem-sheaths is the most important. These findings offer an effective N management strategy for 4R nutrient stewardship (right source, right method, right rate and right timing) of mechanical direct-seeding hybrid *indica* rice.

## 1 Introduction

China is currently undergoing critical transformation from traditional to modern agriculture (Cheng et al., 2023). Although the mechanization rate for plowing and harvesting is high, the rice (*Oryza sativa* L.) machine planting segment lags behind at a rate of less than 50% (National Bureau of Statistics of China, 2022). To improve this situation, China has increased its support for mechanical transplanted rice. However, although machine transplanting technology has significant advantages over traditional seedling raising and hand transplanting, the laborious process of seedling raising and management for mechanical transplanted rice, coupled with the high labor intensity of centralized seedling transport and transplanting, still results in high overall cost (Zhong et al., 2021; He et al., 2023). For large-scale production, direct seeding using rice machines is the most convenient method for rice cultivation (Guo et al., 2023a). Mechanical direct-seeding eliminates the need for raising, transporting, and transplanting seedlings, resulting in improved production efficiency (Farooq et al., 2011). This approach also boosts mechanized planting and is an efficient method for large-scale rice production (Farooq et al., 2011; Yang et al., 2023). However, rice varieties suitable for mechanical direct-seeding in China’s major rice-producing regions are not appropriate (Sun et al., 2022; Guo et al., 2023a). To achieve high-yield and high-efficiency cultivation, it is further essential to integrate agricultural machinery and agronomy deeply (Yang et al., 2022). However, research on the theory of high-quality and high-yield cultivation of mechanically direct-seeding rice is still relatively inadequate.

Nitrogen (N) fertilizers are key for boosting rice production. However, N fertilizers are prone to volatilization and leakage. In China, the N use efficiency (NUE) of rice is low at approximately 30–35%, which is considerably lower than the global average in developed countries (46%) (Peng et al., 2009; Sun et al., 2023a). The primary cause is excessive fertilizer application, which results in diminishing returns. Nitrification and denitrification are significant contributors to low NUE (Chen et al., 2022). To address these issues, measures such as the 4R nutrient stewardship concept (right source, right rate, right time, right place) (IPNI, 2012) promoted by the International Plant Nutrition Institute (IPNI), N fertilizer management (Yokamo et al., 2023; Sun et al., 2023a; Sun et al., 2023b), soil testing and formulated fertilizer application (Chen et al., 2021), and leaf color diagnosis of SPAD meter (Peng et al., 1996) have been implemented by numerous scholars, resulting in positive outcomes. It is crucial to adopt practices to ensure that the N release rate aligns with the crop fertilizer requirements. The development and application of controlled-release fertilizers have garnered increased interest and research due to their potential to enhance N use and production efficiency, while saving time and labor (Ke et al., 2018; Lyu et al., 2021a; He et al., 2023). This area remains a focus of research, with the literature predominantly focusing on hand- and machine-transplanted rice (Ke et al., 2018; Lyu et al., 2021a; Yu et al., 2022). However, there is limited research on the effects of controlled-release fertilizers in mechanically direct-seeding rice. This study primarily examined the various types, optimal application amounts, and methods of side deep fertilization, as well as the use of slow-mixed fertilizers and other relevant factors related to slow-controlled-release fertilizers (Ke et al., 2018; Wu et al., 2021; Sun et al., 2023b). These factors were investigated under a onetime basal application. However, inconsistencies were observed in the study due to variations in controlled-release fertilizers (Wu et al., 2021; Lyu et al., 2021a), nutrient release timing (Cheng et al., 2022), and supporting application techniques (Hou et al., 2021; He et al., 2023). During the later stages, the rate and intensity of controlled-release fertilizers did not meet the immediate requirements of the heavy panicle hybrid rice and super rice varieties. These varieties require increased tillers, grains per panicle, 1000-grain weight, and single panicle weight (Jiang et al., 2016; Sun et al., 2022; Li et al., 2023). Currently, there is limited research on the optimal period for applying N fertilizer during the grain-filling stage of mechanical direct-seeding of heavy panicle hybrid rice. It is uncertain whether adjusting the timing of N fertilizer topdressing to match the growth and development characteristics of the plant will cause improved yield, NUE, and rice quality compared with the basal application of a controlled-release fertilizer.

Based on our previous research (Sun et al., 2022; Guo et al., 2023a; Sun et al., 2023b), we selected heavy panicle super-hybrid rice varieties for this study. We investigated the effects of base application of slow-mixed fertilizer and topdressing of conventional N fertilizer on photosynthetic production and N-use characteristics under the condition of mechanical direct seeding. This study systematically examined the yield and quality of direct-seeding rice, along with its physiological mechanisms. This study proposes an optimal management mode for combining controlled-release N fertilizers with mechanical direct-seeding rice. It also suggests technical regulations for improving quality, yield, and NUE. These findings provide a theoretical and practical basis for implementing high-quality, high-yield, and high-efficiency 4R nutrient stewardship (right source, right method, right rate and right timing) technologies for heavy panicle mechanical direct-seeding hybrid *indica* rice.

## 2 Materials and methods

### 2.1 Study site and materials

**Figure 1:**
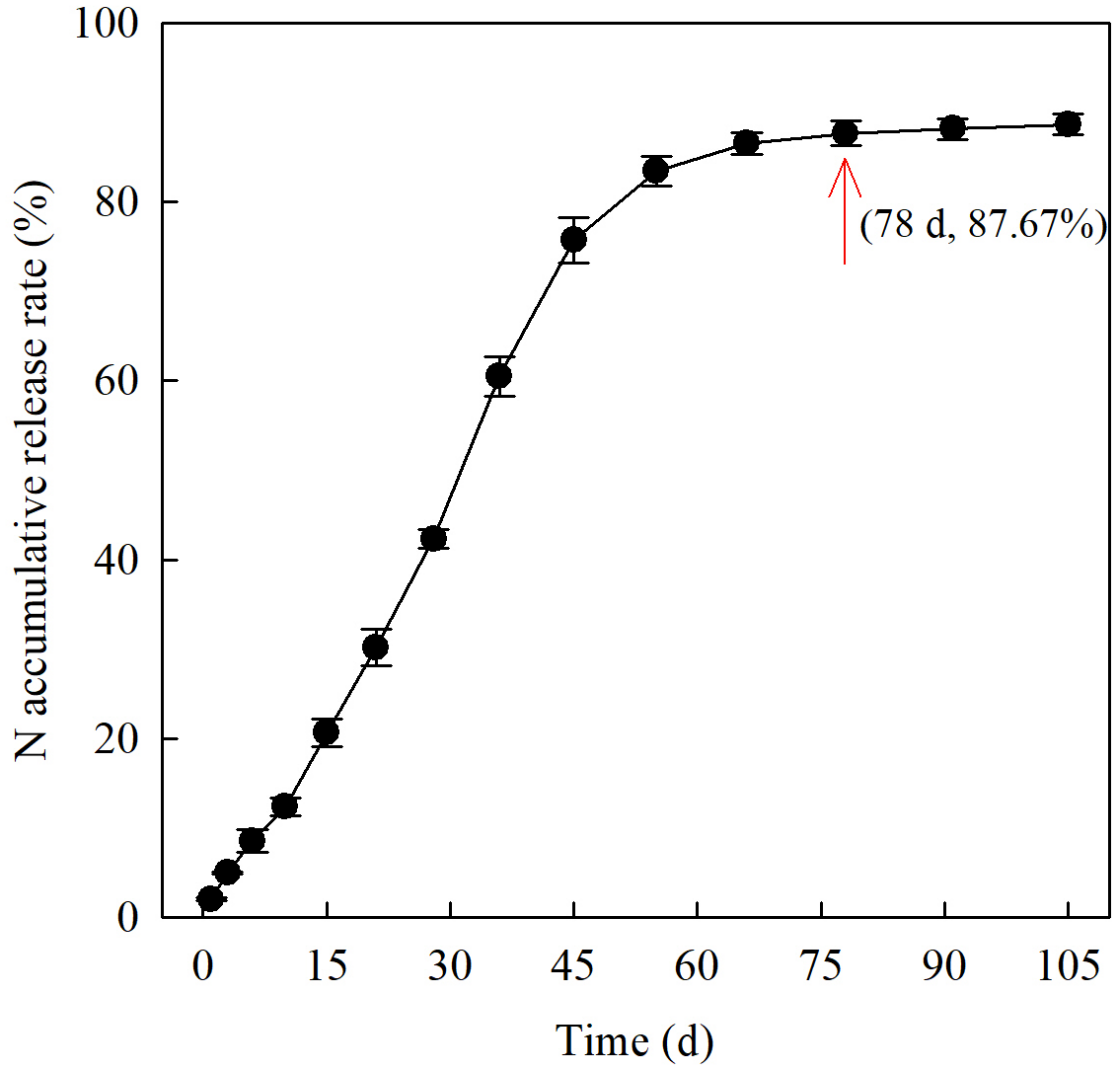
N cumulative release rate curve of controlled-release N fertilizer.

Field experiments were conducted in Chongzhou (103°38′E, 30°33′N), Sichuan Province, China, in 2021 and 2022. The soils samples (0–20 cm) were analyzed physicochemical characteristics before initiation of the experiments (**Table 1**). The study site has a subtropical monsoon humid climate, and the rainfall, sunshine hours, and average temperature, during the rice growing season (May to October) were 924.10 mm, 836.05 h, and 22.77°C in 2021 and 894.20 mm, 762.41 h, and 23.02°C in 2022, respectively. This study used Yixiangyou 2115 (growth periods 145.2 d, female parent Yixiang 1A and male parent Yahui 2115) and Fyou 498 (growth periods 144.5 d, female parent FS3A and male parent Shuhui 498), two representative hybrid *indica* rice cultivars bred by Sichuan Agricultural University that are widely planted in South China. A slow-mixed basal fertilizer was applied, including 120 kg hm^-2^ N fertilizer comprising polymer-coated controlled-release urea-N (90 kg hm^-2^) and conventional urea-N (30 kg hm^-2^), 75 kg hm^-2^ P_2_O_5_, and 150 kg hm^-2^ K_2_O, provided by Yantai Longdeng Fertilizer Co., Ltd. The N-release characteristics of the controlled-release N immersed in water at pH 7 and 25°C and the cumulative release rate of N reached 87.67% within 78 d shown in **Figure 1** as described by Tomaszewska and Jarosiewicz, 2002.

**Table 1:** Average values for selected soil characteristics of composite topsoil samples in the field experiments.

Year	Organic matter (g kg^-1^)	Total N (g kg^-1^)	Available nutrient (mg kg^-1^)			pH	Bulk density (g cm^-3^)
			N	P	K		
2021	18.63	1.48	90.51	21.46	111.72	6.39	1.27
2022	19.64	1.52	89.71	21.98	104.35	6.42	1.28

### 2.2 Experimental design and field management

The experiments used a randomized design with two varieties and four strategies for managing N fertilizer. The comprehensive N fertilizer management mode (**Table 2**) were as follows: (1) slow-mixed N fertilizer (120 kg hm^-2^) as a base (N_1_, as control, in order to further calculate the NUE of urea-N topdressing); (2) N_1_+urea-N (30 kg hm^-2^) once as a base (N_2_); (3) N_1_+urea-N (30 kg hm^-2^) topdressing on 32d after sowing at the tillering stage (N_3_); (4) N_1_+urea-N (30 kg hm^-2^) topdressing on 93d after sowing at the booting stage (N_4_). Rice seeds were sown directly using a 2BDS-6 hand-held rice precision hill-direct-seeding machine (Guilin High-tech Zone Kefeng Machinery Co., Ltd.) on May 14th for both years. The row spacing and plant spacing were 25 cm and 20 cm, respectively, with a sowing amount of 30.0 kg hm^-2^ (4–6 seeds per hole) and density of 200,000 holes hm^-2^. Each treatment had three replicates, with a plot area of 40.8 m^2^ (8.5 m length and 4.8 m width). To prevent water and fertilizer from mixing, the plastic film was wrapped around ridges (40 cm wide and 30 cm high) constructed between the plots. All treatments used a high-efficiency alternation-irrigation technique (Sun et al., 2012). Chemical pesticides have been used to prevent yield loss and experimental errors caused by insects, diseases, and weeds.

**Table 2:** The comprehensive N fertilizer management mode (kg hm^-2^).

Treatments	Total N amount	Basal N fertilizer (1d before sowing)		N topdressing of conventional urea	
		Slow-mixed N	Conventional urea N	Tiller fertilizer (32d after sowing)	Booting fertilizer (93d after sowing)
N_1_	120	120	0	0	0
N_2_	150	120	30	0	0
N_3_	150	120	0	30	0
N_4_	150	120	0	0	30

N_1_: slow-mixed N fertilizer (120 kg hm^-2^) as a base; N_2_: N_1_+urea-N (30 kg hm^-2^) one-time as a base; N_3_: N_1_+urea-N (30 kg hm^-2^) topdressing at tillering stage (32d after sowing); N_4_: N_1_+urea-N (30 kg hm^-2^) topdressing at booting stage (93d after sowing).

### 2.3 Measurement terms and methods

#### 2.3.1 Leaf area index

At the jointing, heading, and maturity stages, we obtained five holes from the representative rice plants in each plot based on the average number of tillers. We measured the leaf area of the rice plants at each growth stage using a CID-203 leaf area analyzer (CID Company, USA). The leaf area index (LAI) was calculated using the method reported by Liu et al. (2022).

#### 2.3.2 Biomass accumulation

Five holes were sampled from each plot, representing rice plants with average tillers at the heading and maturity stages. The samples were divided into four parts: the stem sheaths, leaves, panicles, and roots. The samples were then exposed to 105°C for 40 min and subsequently dried at 80°C until they reached a constant weight (Guo et al., 2023a). The total biomass accumulation was calculated as the sum of the dry matter accumulation of the four plant parts.

#### 2.3.3 Root vigor

As mentioned in Section 2.3.2, the method of Ramasamy et al. (1997) was used. Fresh roots (2.0 g) from each plot sample were transferred into a 100 mL flask. Then, 25.0 mL of 50.0 mg L^-1^α-NA and 25.0 mL of phosphate buffer (0.1 mol L^-1^, pH 7.0) were added. After filtration for 2h at 25°C in a closed shaker, 2.0 mL of NaNO_2_ (100.0 mg L^-1^) and 1.0mL of sulfonamide (1.0%) were added to the filtrate. Color was determined using a Shimadzu-1700 spectrophotometer (Japan) at 510 nm. The results were expressed as mg α-NA g^-1^DW h^-1^.

#### 2.3.4 Nitrogen content

As stated in Section 2.3.2, each part was crushed and sieved separately through an 80-mesh sieve. The N content of each part was determined using the Kjeldahl method (Kjeltec-8400; FOSS, Hillerd, Denmark), as described by Yoshida et al. (1976).

#### 2.3.5 Grain yield and its components

The number of effective panicles per plant was determined by examining 50 holes in rice plants in each plot at maturity. From each plot, 10 holes of rice plants were selected based on the average tillers to examine the total grains per panicle, number of full grains, and 1000-grain weight. The seed-setting percentages were also calculated (Guo et al., 2023a). The grain yield was determined by harvesting each 12.0 m² plot without border plants and adjusting it to a standard moisture content of 13.5% using a grain moisture meter (PM-8188-A, Kett Electric Laboratory, Tokyo, Japan).

#### 2.3.6 Grain quality measurements

Approximately 1000 g of grains were harvested from each plot and naturally dried in the shade for three months to stabilize their physicochemical properties. The grains were then analyzed for quality after being passed through a dehusker and polished. A 250-gram sample was separated into broken and unbroken grains, and the brown rice, milled rice, and head rice rates were expressed as percentages of the total 250-gram rice grains (Zhang et al., 2008). For each sample, 1000 whole milled rice grains were randomly selected and scanned to create a digital image. This process was repeated three times. Image analysis software (JMWT-12, Dongfujiuheng Instrument Technology Co., Ltd., Beijing, China) was used to determine the rates of chalky grains and the degree of chalkiness (Guo et al., 2023b). The taste of cooked rice was measured using a Satake Rice Taste Analyzer (STA1A type, Satake, Japan) (Shi et al., 2022). 30.0 g of milled rice were weighed and placed in a stainless steel tank. Water was added at a rice-to-water ratio of 1:1.4, and the mixture was soaked for 30 min. The mouth of the tank was wrapped with filter paper and the tank was placed in a steam electric rice cooker. The rice was steamed for 30 min and then cooled for 2 h. After cooling, 7.0 g of rice was weighed at 25°C and placed into a special rice press instrument to form a rice cake. The rice cakes were then placed in a taste analyzer for testing.

### 2.4 Indicator calculation

As mentioned in Section 2.3 measurement terms, the calculation and definition of the following parameters are based on the method of Sun et al. (2023a).

Population photosynthetic potential from jointing to heading stage (PP; ×10^4^ m^2^ d hm^-2^) = 1/2 (the leaf area at the jointing stage + the leaf area at the heading stage) × (the time at the heading stage-the time at the jointing stage)
Dry matter transport rate in stem sheaths (DTR; %) = (dry matter weight in stem sheaths at the maturity stage-dry matter weight in stem sheaths at the heading stage)/dry matter weight in stem sheaths at the heading stage × 100
Dry matter transport contribution rate in stem sheaths (DCR; %) = (dry matter weight in stem sheaths at the maturity stage-dry matter weight in stem sheaths at the heading stage)/grain weigh at the maturity stage × 100
Population growth rate (PGR; g·m^-2^·d^-1^) = (dry matter weight in plants at the maturity stage-dry matter weight in plants at the heading stage)/(the time at the maturity stage-the time at the heading stage)
Root vigor of decay rate from heading to maturity stage (DCRT; %) = (root vigor at the maturity stage-root vigor at the heading stage)/root vigor at the heading stage ×100
N transport amount in leaves or stem sheaths from heading to maturity stage (NTA; kg hm^-2^) = N accumulation amount in leaves or stem sheaths at the heading stage-N accumulation amount in leaves or stem sheaths at the maturity stage
N transport rate in leaves or stem sheaths from heading to the maturity stage (NTR; %) = NTA in leaves or stem sheaths/N accumulation amount in leaves or stem sheaths at the heading stage
N transport contribution rate in leaves or stem sheaths from heading to the maturity stage (NCR; %) = NTA in leaves or stem sheaths/N accumulation amount in panicles at the maturity stage
N agronomic efficiency of urea-N topdressing (NAE; kg kg^-1^) = (grain yield in urea-N topdressing supply-grain yield in zero urea-N topdressing supply)/urea-N topdressing supply amount
N recovery efficiency of urea-N topdressing (NPE; kg kg^-1^) = (grain yield in urea-N topdressing supply-grain yield in zero urea-N topdressing supply)/(total N accumulation in urea-N topdressing supply at the maturity stage-total N accumulation in zero urea-N topdressing supply at the maturity stage)

### 2.5 Statistical analysis

Data were analyzed using Microsoft Excel 2010. Analysis of variance (ANOVA) was performed using the statistical program SPSS 18.0 (SPSS Statistics, SPSS Inc., Chicago, IL, USA). Graphs were generated using SigmaPlot 10.0 (Systat Software Inc., Chicago, IL, USA). The treatment means were tested using the least significant difference (LSD) test (*P* < 0.05). Principal component analysis was conducted using Origin 2021 (OriginLab Corp., Northampton, MA, USA).

## 3 Results

### 3.1 Yield and yield components

The impact of varying N fertilizer management on grain yield and its components in mechanical direct-seeding hybrid *indica* rice is significant. The interaction effect of the two factors on grain yield, filled spikelets, total spikelets, and seed-setting rate was also significant. The trend observed in the two-year experiments was consistent (**Table 3**). Under different varieties and N fertilizer management, the impact of N fertilizer on yield was significantly greater than variety. The yields of the N_2_, N_3_, and N_4_ treatments increased by 4.45%, 13.29%, and 20.98%, respectively, compared to the N_1_ treatment, with the highest yield observed in the N_4_ treatment. Fyou 498 showed a greater increase under N_2_ and N_3_ treatments compared to Yixiangyou 2115. This suggests that varieties with a large panicle type and high sink capacity, such as Fyou 498, should be treated with late booting fertilizer besides the base application of slow-mixed fertilizer to exploit their high-yield potential.

**Table 3:** Effects of slow-mixed fertilizer base application combined with urea topdressing time on yield and its components of mechanical direct-seeding hybrid *indica* rice.

Year	Cultivar	N treatments	Effective panicles (×10^4^·hm^-2^)	Filled spikelets (No. panicle^-1^)	Total spikelets (×10^6^·hm^-2^)	Filled grains (%)	1000-grain weight (g)	Grain yield (kg·hm^-2^)
2021	Yixiangyou2115	N_1_	229.83c	116.60c	267.94c	81.14b	36.00b	8100.85c
		N_2_	239.00b	122.15bc	291.15b	78.67c	36.06b	8458.75c
		N_3_	252.66a	128.10b	323.73a	81.95b	36.06b	9169.85b
		N_4_	234.23bc	140.00a	327.94a	83.55a	36.87a	9825.54a
	**Average**		**238.93**	**126.71**	**302.70**	**81.33**	**36.25**	**8888.75**
	Fyou 498	N_1_	220.00c	145.28c	319.74c	81.53c	32.06ab	8297.75c
		N_2_	228.70b	150.06bc	343.15b	80.67c	31.66b	8746.28c
		N_3_	236.80a	156.44b	370.36a	83.25b	31.93ab	9494.78b
		N_4_	227.61bc	167.41a	381.05a	84.83a	32.18a	10090.28a
	**Average**		**228.28**	**154.80**	**353.58**	**82.57**	**31.96**	**9157.27**
	*F* value	V	54.91**	367.03**	113.51**	19.03**	4457.63**	5.16*
		N	33.28**	44.67**	34.33**	42.43**	13.39**	43.57**
		V×N	1.74	6.04*	4.92*	4.23*	2.23	4.45*
2022	Yixiangyou2115	N_1_	223.02c	114.44c	267.79c	76.12bc	35.33b	8004.75c
		N_2_	234.00b	121.04b	269.94c	74.18c	35.60b	8239.32c
		N_3_	257.67a	122.52b	299.60b	79.76b	36.25ab	8949.53b
		N_4_	234.17b	127.94a	315.69a	84.73a	36.88a	9556.51a
	**Average**		**237.21**	**121.48**	**288.25**	**78.70**	**36.01**	**8687.52**
	Fyou 498	N_1_	220.00c	144.21c	329.75c	72.33b	32.05b	8075.86d
		N_2_	228.67b	146.11c	346.03b	70.76b	32.13ab	8481.72c
		N_3_	236.83a	152.23b	354.35a	79.79a	32.15ab	9187.14b
		N_4_	233.57ab	161.07a	355.57a	80.52a	32.49a	9825.58a
	**Average**		**229.77**	**150.90**	**346.42**	**75.85**	**32.20**	**8892.58**
	*F* value	V	54.69**	52.04**	31.46**	50.71**	412.88**	6.99*
		N	33.17**	21.64*	6.59*	49.24**	6.77*	38.78**
		V×N	1.77	7.02*	5.21*	6.47*	1.25	7.15*

N_1_: slow-mixed N fertilizer (120 kg hm^-2^) as a base; N_2_: N_1_+urea-N (30 kg hm^-2^) one-time as a base; N_3_: N_1_+urea-N (30 kg hm^-2^) topdressing at the tillering stage (32d after sowing); N_4_: N_1_+urea-N (30 kg hm^-2^) topdressing at the booting stage (93d after sowing). V, Variety; N, N fertilizer treatment; V×N, cultivar and N fertilizer treatment interaction.*, *P* < 0.05; **, *P* < 0.01. Different lowercase letters indicate significant (*P* < 0.05) differences among N fertilizer treatments under the same variety.

**Table 3** show that N fertilizer management resulted in higher numbers of filled spikelets, total spikelets, and filled grains than the effects of variety differences. However, the number of effective panicles and 1000-grain weight showed opposite trends. This suggests that a suitable combination of variety and N fertilizer regulation can adjust the yield components of mechanical direct-seeding hybrid *indica* rice, ultimately promoting yield. The mean values of the yield components for Fyou 498 were significantly higher than those for Yixiangyou 2115 among the different varieties, except for the number of effective panicles and 1000-grain weight. Under the application of slow-release fertilizer combined with urea-N topdressing, the number of effective panicles initially increased and then decreased with a delay in the urea-N fertilizer application time. The yield components of different varieties also increased with the delay in the urea-N fertilizer application time. The filled spikelets, total spikelets, filled grains, and 1000-grain weight of the two varieties under N_4_ treatment were higher than those under the other treatments. This compensated for the deficiency of the effective panicle number and was a significant factor in achieving a high yield under N_4_ treatment.

### 3.2 Leaf area index and population photosynthetic potential

**Figure 2:**
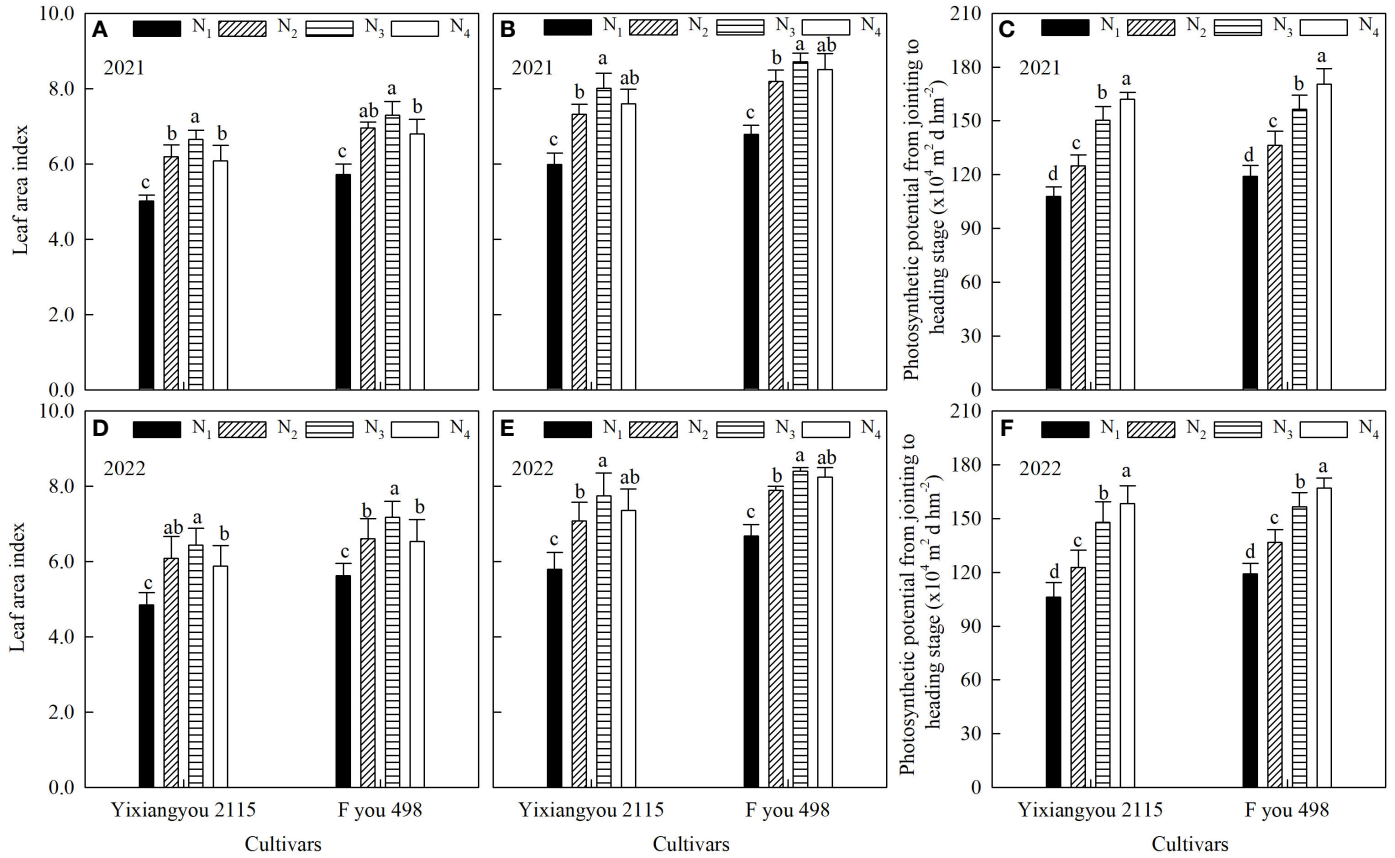
Effects of slow-mixed fertilizer base application combined with urea topdressing time on LAI at the jointing stage **(A, D)**, LAI at the heading stage **(B, E)** and photosynthetic potential **(C, F)** from the jointing to heading stage of mechanical direct-seeding hybrid *indica* rice. N_1_: slow-mixed N fertilizer (120 kg hm^-2^) as a base; N_2_: N_1_+urea-N (30 kg hm^-2^) one-time as a base; N_3_: N_1_+urea-N (30 kg hm^-2^) topdressing at the tillering stage (32 d after sowing); N_4_: N_1_+urea-N (30 kg hm^-2^) topdressing at the booting stage (93 d after sowing). Different lowercase letters indicate significant (*P* < 0.05) differences among N fertilizer treatments under the same variety.

At both the jointing and heading stages, the LAI of Fyou 498 was significantly higher than Yixiangyou 2115 by 9.61–13.91% and 8.67–13.35%, respectively. This trend was consistent across both years of experimentation (**Figures 2A, B, D, E**). For the same variety under different N fertilizer management, LAI at the jointing and heading stages showed an initial increase, followed by a decrease. Compared to the N_1_ treatment, the average values of the N_2_, N_3_, and N_4_ treatments for the two varieties during the jointing stage (N_4_ treatment without topdressing urea-N fertilizer during this time) were significantly higher by 22.62%, 30.15%, and 20.63%, respectively. At the heading stage, the LAI of the N_2_, N_3_, and N_4_ treatments increased significantly by 21.45%, 31.03%, and 26.20%, respectively. The LAI in the N_4_ treatment showed the most significant increase. From the jointing to heading stage, the population photosynthetic potential of Fyou 498 was 5.01%–10.49% higher than Yixiangyou 2115 (**Figures 2C, F**). The photosynthetic potential of the population under the same variety increased significantly from the jointing to the heading stage with the postponement of urea-N fertilizer application. Compared with the N_1_ treatment, the N_2_, N_3_, and N_4_ treatments increased significantly by 15.07%, 35.43%, and 47.09%, respectively.

### 3.3 Dry matter accumulation and translocation and population growth rate

The impact of variety and N fertilizer on the indices of dry matter accumulation and transport, as well as the population growth rate from the heading to the maturity stage, was significant. The interaction effect of the two factors on the amount of dry matter accumulation, transport rate, and contribution rate in stem sheaths, as well as the population growth rate, was also significant (**Table 4**). Furthermore, the accumulation and translocation of dry matter, as well as the population growth rate from the heading to maturity stage, were significantly more affected by the management of N fertilizer than by the differences between the varieties. The amount of dry matter accumulated in the stem sheaths of Fyou 498 was 4.46% higher than Yixiangyou 2115 at the heading stage, and 2.42% higher at the maturity stage. The dry matter accumulation, dry matter transport rate, dry matter transport contribution rate, and population growth rate of Fyou 498 from the heading to maturity stage were 8.83%, 5.48%, 7.09%, and 8.40% higher than those of Yixiangyou 2115, respectively. The indices of dry matter accumulation and population growth rate increased to varying degrees with the postponement of the urea-N topdressing time under the same variety. Compared to the N_1_ treatment, the N_2_, N_3_, and N_4_ treatments significantly increased the dry matter accumulation in stem sheaths during the heading and maturity stages by 3.04–40.43% and 17.31–53.50%, respectively. Furthermore, the dry matter accumulation in plants and population growth rate from the heading to maturity stage were significantly increased by 27.18–78.55% and 26.69–62.72%, respectively. Compared with the N_1_ treatment, the dry matter transport rate and contribution rate in stem sheaths from heading to maturity among the N_2_, N_3_, and N_4_ treatments were significantly reduced by 6.95–8.77% and 2.57–6.18%. However, the dry matter transport rate and contribution rate in stem sheaths from heading to maturity increased by 1.18–1.82% and 3.11–3.62% with the postponement of the urea-N fertilizer application time in the N_2_, N_3_, and N_4_ treatments.

**Table 4:** Effects of slow-mixed fertilizer base application combined with urea topdressing time on dry matter accumulation and translocation and population growth rate from the heading to maturity stage of mechanical direct-seeding hybrid *indica* rice.

Cultivar	N treatments	DASH (kg·hm^-2^)	DASM (kg·hm^-2^)	From heading to maturity stage			
				DAP (kg·hm^-2^)	DTR (%)	DCR (%)	PGR (g·m^-2^·d^-1^)
Yixiangyou 2115	N_1_	5900.27b	3899.34d	3002.20d	33.91a	24.69a	10.00d
	N_2_	6079.29b	4574.95c	3818.40c	24.75c	17.78c	12.73c
	N_3_	7453.23a	5545.42b	4564.10b	25.60b	20.81b	15.21b
	N_4_	7849.36a	5800.19a	5012.60a	26.11b	20.86b	16.71a
**Average**		**6820.54**	**4954.98**	**4099.33**	**27.59**	**21.04**	**13.66**
Fyou498	N_1_	5948.65d	3904.58d	3146.80d	34.36a	24.71a	10.49d
	N_2_	6480.93c	4797.13c	3986.30c	25.98d	19.25d	13.29c
	N_3_	7749.27b	5618.85b	5122.10b	27.49c	22.44c	17.07b
	N_4_	8354.37a	5993.65a	5618.90a	28.26b	23.40b	18.73a
**Average**		**7133.31**	**5078.55**	**4468.53**	**29.02**	**22.45**	**14.90**
*F* value	V	13.17**	3.98*	47.47**	16.77**	27.11**	47.79**
	N	145.77**	210.84**	346.57**	128.43**	89.13**	348.01**
	V×N	1.29	0.67	5.31**	4.82*	4.10*	5.29**

N_1_: slow-mixed N fertilizer (120 kg·hm^-2^) as a base; N_2_: N_1_+urea-N (30 kg·hm^-2^) one-time as a base; N_3_: N_1_+urea-N (30 kg·hm^-2^) topdressing at the tillering stage (32d after sowing); N_4_: N_1_+urea-N (30 kg·hm^-2^) topdressing at the booting stage (93d after sowing). V, Variety; N, N fertilizer treatment; V×N, cultivar and N fertilizer treatment interaction. DASH, dry matter accumulation amount in stem-sheath at the heading stage; DASM, dry matter accumulation amount in stem sheaths at the maturity stage; DAP, dry matter accumulation amount in plants; DTR, dry matter transport rate in stem sheaths; DCR, dry matter transport contribution rate in stem sheaths; PGR, population growth rate. *, *P* < 0.05; **, *P* < 0.01. Different lowercase letters indicate significant (*P* < 0.05) differences among N fertilizer treatments under the same variety.

### 3.4 Root vigor

**Figure 3:**
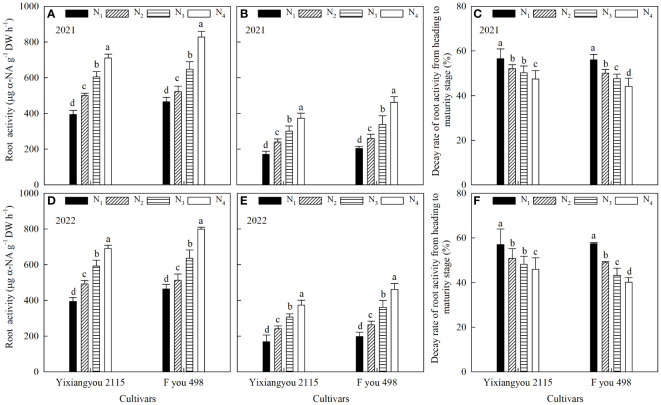
Effects of slow-mixed fertilizer base application combined with urea topdressing time on root vigor at the heading stage **(A, D)**, root vigor at the maturity stage **(B, E)** and root vigor decay rate **(C, F)** from the heading to maturity stage of mechanical direct-seeding hybrid *indica* rice. N_1_: slow-mixed N fertilizer (120 kg hm^-2^) as a base; N_2_: N_1_+urea-N (30 kg hm^-2^) one-time as a base; N_3_: N_1_+urea-N (30 kg hm^-2^) topdressing at the tillering stage (32 d after sowing); N_4_: N_1_+urea-N (30 kg hm^-2^) topdressing at the booting stage (93 d after sowing). Different lowercase letters indicate significant (*P* < 0.05) differences among N fertilizer treatments under the same variety.

The root vigor of Fyou 498 was significantly higher than Yixiangyou 2115 at both the heading (**Figures 3A, D**) and maturity stages (**Figures 3B, E**), with differences ranging from 4.80% to 18.15% and 8.76% to 23.88%, respectively. Furthermore, the root vigor decay rate from the heading to maturity stage (**Figures 3C, F**) was 1.76–7.08% lower in Fyou 498 than in Yixiangyou 2115. The results of the 2-year experiments showed consistent trends. In the same variety, postponing the application time of urea-N resulted in a significant increase in root vigor during the heading and maturity stages. Compared with the N_1_ treatment, the root vigor at the heading stage increased significantly by 12.05–27.04%, 39.07–53.36%, and 77.89–80.09% in the N_2_, N_3_, and N_4_ treatments, respectively. At the maturity stage, root vigor increased significantly by 27.60–40.42%, 65.91–76.26%, and 118.52–126.18% in the N_2_, N_3_, and N_4_ treatments, respectively. Moreover, the decay rate of root vigor from the heading to maturity stage decreased with postponement of the urea-N fertilizer application time. Compared to the N_1_ treatment, root vigor decay rate decreased significantly by 7.91–10.72%, 11.05- 15.07%, and 16.05–21.39% in the N_2_, N_3_, and N_4_ treatments, respectively, from the heading to maturity stage.

### 3.5 Nitrogen transport and utilization

**Table 5** shows that the effect of variety on the N transport contribution rate in stem sheaths from the heading to the maturity stage was not significant. However, the effects of variety and N fertilizer management on N translocation in stem sheaths (leaves) from heading to maturity and NUE (NAE and NPE) were significant. Furthermore, the interaction effect of these two factors on N transport in stem sheaths and leaves, as well as the transport rate from heading to maturity, was significant. Compared to Yixiangyou 2115, Fyou 498 exhibited an increase of 1.03–9.38% in the N transport rate and N transport contribution rate in stem sheaths from heading to maturity. Moreover, Fyou 498 showed an increase of 3.34–12.01% in the N transport rate and N transport contribution rate in leaves from heading to maturity. The NAE and NPE of Fyou 498 were significantly higher than those of Yixiangyou 2115, by 13.98% and 15.44%, respectively. For the same variety, the amount of N transported in stem sheaths (leaves) and the contribution rate of N transport in stem sheaths (leaves) increased to varying degrees from the heading to maturity stage, with a delay in the urea-N topdressing time. Compared to the N_1_ treatment, the N_2_, N_3_, and N_4_ treatments significantly increased the amount of N transported in stem sheaths (leaves) from the heading to maturity stage by 13.54–59.96%. Furthermore, the N transport contribution rate in stem sheaths (leaves) increased by 0.29–16.50%. However, the rate of N transport in stem sheaths (leaves) from the heading to maturity stage decreased by 2.13–25.27% when the urea-N topdressing was postponed. Moreover, both NAE and NPE increased significantly with a delay in the urea-N topdressing.

**Table 5:** Effects of slow-mixed fertilizer base application combined with urea topdressing time on N translocation in stem-sheaths and leaves from the heading to maturity stage and NUE of mechanical direct-seeding hybrid *indica* rice.

Cultivar	N treat-ments	NSTA (kg·hm^-2^)	NSTR (%)	NSCR (%)	NLTA (kg·hm^-2^)	NLTR (%)	NLCR (%)	NAE (kg kg^-1^)		NPE (kg kg^-1^)	
								2021	2022	2021	2022
Yixiangyou 2115	N_1_	10.37d	28.88a	10.04b	28.35d	69.08a	27.45b	–	–	–	–
	N_2_	11.94c	26.39b	10.22b	32.19c	53.88b	27.57b	11.93c	7.82c	9.91c	6.50c
	N_3_	14.40b	26.16bc	10.86a	36.76b	52.63bc	27.74b	35.63b	31.49b	16.52b	14.60b
	N_4_	16.25a	25.06c	11.60a	39.70a	51.62c	28.33a	57.49a	51.73a	20.43a	18.38a
**Average**		**13.24**	**26.62**	**10.68**	**34.25**	**56.80**	**27.77**	**35.02**	**30.35**	**15.62**	**14.16**
Fyou 498	N_1_	11.34d	30.03a	10.18c	30.74d	69.64a	27.60c	–	–	–	–
	N_2_	12.95c	29.39a	10.21c	36.24c	57.30b	28.56b	14.95c	13.53c	13.13c	11.88c
	N_3_	15.50b	26.86b	10.91b	41.44b	56.42bc	29.18a	39.90b	37.04b	18.40b	17.08b
	N_4_	18.14a	26.77b	11.86a	45.04a	55.25c	29.46a	59.75a	58.32a	20.53a	20.04a
**Average**		**14.48**	**28.26**	**10.79**	**38.36**	**59.65**	**28.70**	**38.20**	**36.30**	**17.35**	**16.33**
*F* value	V	52.03**	19.95**	0.68	83.93**	15.76**	4.08*	46.81**	40.21**	53.11**	65.37**
	N	261.91**	23.76**	31.54**	156.40*	87.95**	7.18*	240.87**	163.59**	132.32**	142.57**
	V×N	3.85*	4.34*	0.20	3.98*	3.89*	0.63	4.93*	5.07*	4.44*	4.01*

N_1_: slow-mixed N fertilizer (120 kg·hm^-2^) as a base; N_2_: N_1_+urea-N (30 kg·hm^-2^) one-time as a base; N_3_: N_1_+urea-N (30 kg·hm^-2^) topdressing at the tillering stage (32d after sowing); N_4_: N_1_+urea-N (30 kg·hm^-2^) topdressing at the booting stage (93d after sowing). V: Variety; N: N fertilizer treatment; V×N: cultivar and N fertilizer treatment interaction. NSTA, N transport amount in stem sheaths; NSTR, N transport rate in stem sheaths; NSCR, N transport contribution rate in stem sheaths; NLTA, N transport amount in leaves; NLTR, N transport rate in leaves; NLCR, N transport contribution rate in leaves; NAE, N agronomic efficiency of urea-N topdressing; NPE, N recovery efficiency of urea-N topdressing. *, *P* < 0.05; **, *P* < 0.01. Different lowercase letters indicate significant (*P* < 0.05) differences among N fertilizer treatments under the same variety.

### 3.6 Rice quality

The 2-year experiments showed significant effects of variety and N fertilizer management on rice quality indicators. The interaction effect of the two factors only had a significant impact on chalkiness and chalky kernel rate (**Table 6**). Regarding the treatment of varieties and N fertilizer management, the rice quality indicators of the variety differences were significantly higher than those of N fertilizer management, except for the brown rice rate. Compared with Yixiangyou 2115, Fyou 498 exhibited a decrease in brown rice rate, milled rice rate, head rice rate, and taste value by 0.52–5.51%, 1.53–3.40%, 3.73–6.64%, and 5.05–7.67%, respectively. Furthermore, the chalkiness and chalky kernel rates increased by 1.63–4.91% and 4.37–13.17%, respectively. When comparing the same variety, brown rice, milled rice, head rice, chalkiness, and chalky grain rates increased to varying degrees with the postponement of the urea-N topdressing time. Compared to the N_1_ treatment, the brown rice, milled rice, head rice, chalkiness, and chalky grain rates increased by 0.65–5.09%, 0.67–3.75%, 1.17–6.44%, 0.21–3.48%, and 0.52–12.77% in the N_2_, N_3_, and N_4_ treatments, respectively. Compared to the N_1_ treatment, the taste values of the N_2_, N_3_, and N_4_ treatments decreased significantly by 1.16–5.88%. However, the taste value increased significantly when the urea-N topdressing time was delayed.

### 3.7 Relationships between photosynthetic production, root vigor, N transport, yield, rice quality, and NUE

**Figure 4:**
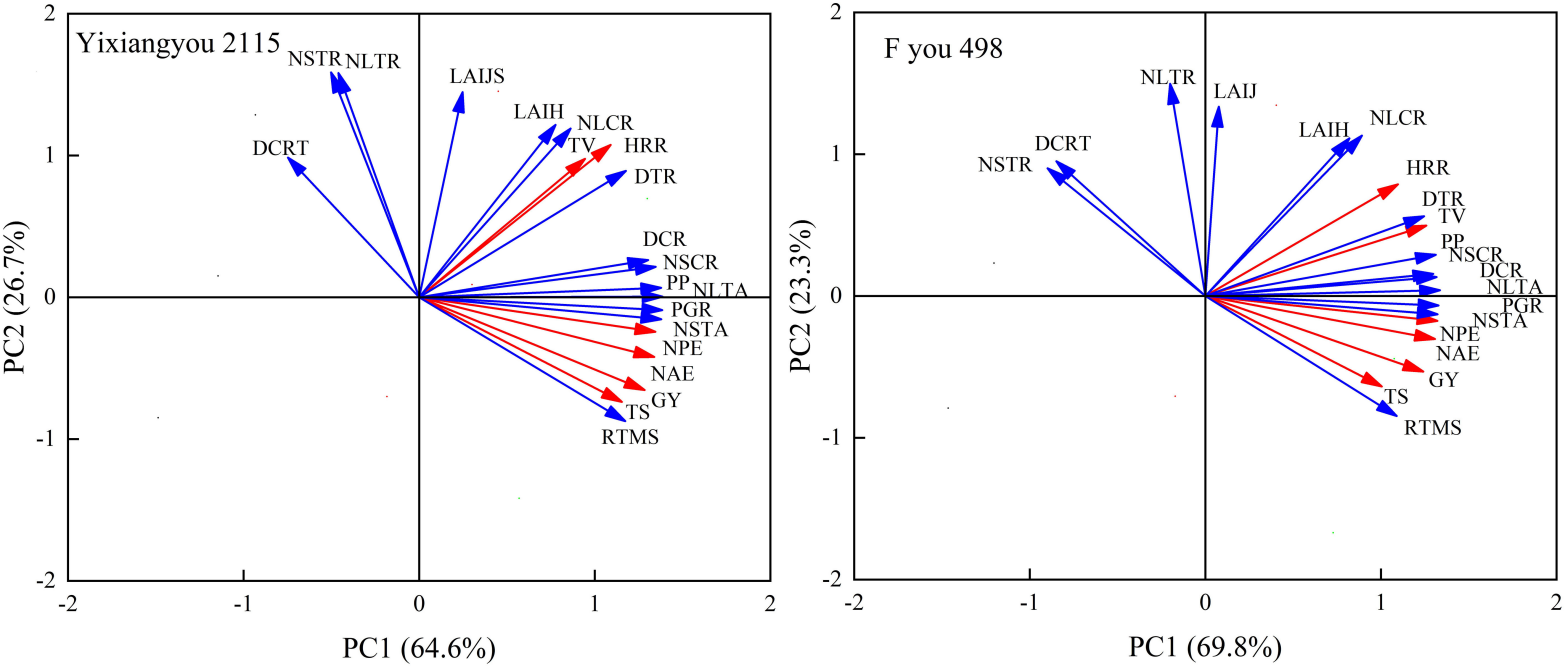
Principal component analysis of grain yield, rice quality and NUE with photosynthetic production, root vigor and N transport. The number of samples is 24 in the analysis of each indicator under every variety. GY, grain yield; TS, total spikelets; NAE, N agronomic efficiency of urea-N topdressing; NPE, N recovery efficiency of urea-N topdressing; HRR, head rice rate; TV, taste value; LAIJ, LAI at the jointing stage; LAIH, LAI at the heading stage; PP, photosynthetic potential from the jointing to heading stage; RTMS, root vigor at the maturity stage; DCRT, root vigor of decay rate from the heading to maturity stage; DTR, dry matter transport rate in stem sheaths from the heading to maturity stage; DCR, dry matter transport contribution rate in stem sheaths from the heading to maturity stage; PGR, population growth rate from heading to maturity stage; NSTA, N transport amount in stem sheaths from the heading to maturity stage; NSTR, N transport rate in stem sheaths from heading to maturity stage; NSCR, N transport contribution rate in stem sheaths from the heading to maturity stage; NLTA, N transport amount in leaves from the heading to maturity stage; NSTR, N transport rate in leaves from the heading to maturity stage; NSCR, N transport contribution rate in leaves from the heading to maturity stage.

Principal component analysis (PCA) was conducted to analyze the relationship between photosynthetic production, root vigor, and N translocation and grain yield, total spikelets, NUE, head rice rate, and taste value under different varieties and N application management (**Figure 4**). Under various N fertilizer treatments, Yixiangyou 2115 and Fyou 498 had principal components 1 and 2, explaining 92.2% and 93.1% of the total changes in grain yield, total spikelets, NAE, NPE, head rice rate, and taste value, respectively. The relationship between these variables suggested that grain yield, NAE, NPE, and taste value of the two varieties were correlated positively with LAI at the jointing stage, LAI at the heading stage, photosynthetic potential from the jointing to heading stage, root vigor at the maturity stage, dry matter transport rate and dry matter transport contribution rate in stem sheaths from the heading to maturity stage, population growth rate from the heading to maturity stage, N transport amount and N transport contribution rate in stem sheaths from the heading to maturity stage, N transport amount and N transport contribution rate in leaves from the heading to maturity stage (**Figure 4**). However, they correlated negatively with the root vigor of decay rate from the heading to maturity stage, N transport rate in stem sheaths from heading to maturity stage, and N transport rate in leaves from heading to maturity stage (**Figure 4**). The total explained amount of the two rice varieties under different N fertilizer treatments (**Figure 4**), compared to Yixiangyou 2115, can better explain the synergistic enhancement of high yield, good quality, and high NUE in rice, including grain yield, rice quality, and NUE for Fyou 498.

**Figure 5:**
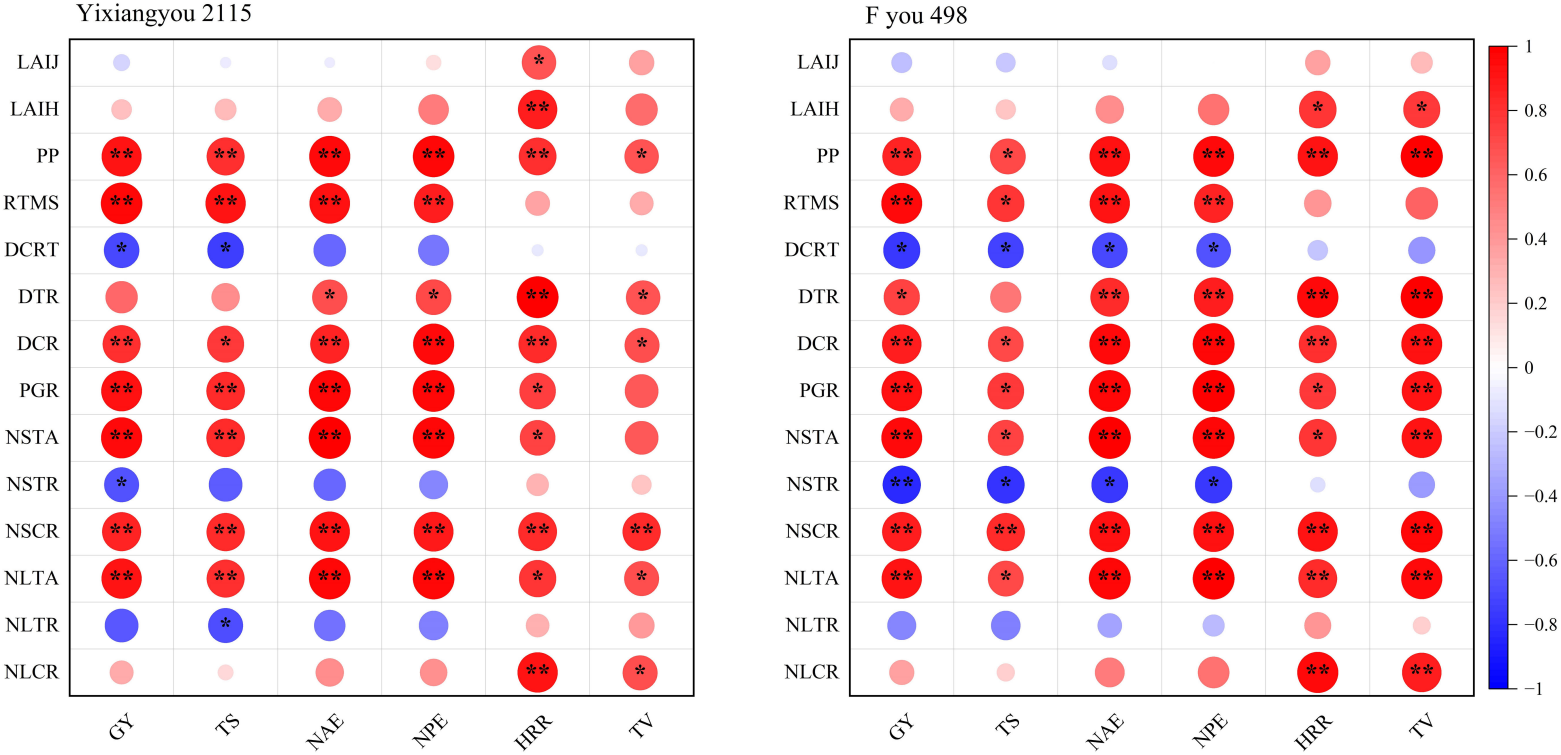
Heat map of person correlation in grain yield, rice quality and NUE with photosynthetic production, root vigor and N transport. The number of samples is 24 in the analysis of each indicator under every variety. GY, grain yield; TS, total spikelets; NAE, N agronomic efficiency of urea-N topdressing; NPE, N recovery efficiency of urea-N topdressing; HRR, head rice rate; TV, taste value; LAIJ, LAI at the jointing stage; LAIH, LAI at the heading stage; PP, photosynthetic potential from the jointing to heading stage; RTMS, root vigor at the maturity stage; DCRT, root vigor of decay rate from the heading to maturity stage; DTR, dry matter transport rate in stem sheaths from the heading to maturity stage; DCR, dry matter transport contribution rate in stem sheaths from the heading to maturity stage; PGR, population growth rate from the heading to maturity stage; NSTA, N transport amount in stem sheaths from the heading to maturity stage; NSTR, N transport rate in stem sheaths from heading to maturity stage; NSCR, N transport contribution rate in stem sheaths from the heading to maturity stage; NLTA, N transport amount in leaves from the heading to maturity stage; NSTR, N transport rate in leaves from heading to maturity stage; NSCR, N transport contribution rate in leaves from the heading to maturity stage. *, *P* < 0.05; **, *P* < 0.01.

The correlation analysis in **Figure 5** indicates that the grain yield, total spikelets, NAE, NPE, head rice rate, and taste value of both varieties were significantly positively correlated with the photosynthetic potential from the jointing to heading stage, dry matter transport contribution rate in stem sheaths from the heading to maturity stage, N transport contribution rate in stem sheaths from the heading to maturity stage, and N transport amount in leaves from the heading to maturity stage. The correlations between the N transport contribution rate in stem sheaths from the heading to maturity stage and the photosynthetic potential from the jointing to heading stage, population growth rate from the heading to maturity stage, and N transport amount in leaves from the heading to maturity stage were highly significant. This indicates that increasing the N transport contribution rate in stem sheaths from the heading to maturity stage can synergistically enhance the high yield, high quality, and high NUE of mechanical direct-seeding hybrid *indica* rice. Furthermore, when considering each variety, the effects of different N fertilizer management on the taste regulation of the high taste value Yixiangyou 2115 were significantly less pronounced than those of the low-taste value Fyou 498. This suggests that there is significant potential for improving the taste value of low-taste value varieties through urea-N topdressing under slow-mixed fertilizer-based application.

## 4 Discussion

### 4.1 Effect of slow-mixed fertilizer base application combined with available N fertilizer on rice yield formation

Selecting appropriate rice varieties and optimizing N fertilizer management are crucial for regulating rice yield (Jiang et al., 2016; Li et al., 2023). The most important factors affecting yield were the number of effective panicles, number of filled spikelets, and 1000-grain weight. Different conclusions have been drawn regarding how to balance the relationships between these factors under varying cultivation conditions (Guo et al., 2023a; Sun et al., 2023a). Previous studies have shown that the productivity of rice varieties primarily depends on the total number of spikelets, which is the product of the number of effective panicles and number of grains per panicle (Wei et al., 2016; Lyu et al., 2021a; Li et al., 2023). The study results indicate that the N_1_ treatment, which involves using urea-N fertilizer as a base fertilizer without topdressing in the later stage and varieties with excessive total spikelets, can lead to issues such as unfilled grains, decreased seed-setting rate, and reduced 1000-grain weight. This is compared to the N_3_ and N_4_ treatments (**Table 3**). Previous studies have suggested that onetime basal application of controlled-release fertilizers and formulations can increase rice yield and efficiency. This method is effective for increasing rice yield and efficiency. This method also regulates the amount of fertilizer used (Deng et al., 2021; Hou et al., 2021; He et al., 2023). However, this study suggests that slow-release fertilizers should be applied as a single basal application. If combined with urea-N in the later stage, the ‘sink’ capacity of the variety should be taken into consideration. In this study, Fyou 498 had a significantly higher total spikelet count than Yixiangyou 2115, but Yixiangyou 2115 had a 1000-grain weight >36.0 g (grain length 7.60 mm, grain width 2.65 mm, and length-width ratio 2.87 of single grain). The 1000-grain weight indirectly reflects the rice’s ‘sink’ capacity based on a certain amount of total spikelets. Both varieties in this study had a large ‘sink’ capacity. Therefore, to promote a significant increase in grain yield (**Table 3**), it is necessary to consider the combined application of urea-N fertilizer in the later stage, based on the application of slow-mixed fertilizer.

Rice yield is closely related to the dynamics of population tillers, photosynthetic characteristics, material accumulation, and transport capacity (Sun et al., 2012; Guo et al., 2023a). Super-high-yield rice is characterized by a lower number of tillers in the early growth stage but a higher percentage of productive tillers. LAI and dry matter accumulation exhibited slow growth in the early stage, moderate growth during the heading stage, and a significant increase after the heading stage. The population growth rate was high, and 70–80% of the grain yield was achieved during the late growth stage (Cheng et al., 2022; Liu et al., 2022; Li et al., 2023). The study showed that using slow-mixed fertilizer combined with urea-N fertilizer N_3_ treatment can increase the photosynthetic potential from the jointing to heading stage and the root vigor decay rate from the heading to maturity stage. This is important for ensuring a high population growth rate and dry matter quality in the late growth stage, resulting in a high yield and efficiency of direct-seeding rice. These findings further enriched and improved the results of previous studies (Wang et al., 2021; Cheng et al., 2022; Liu et al., 2022; Guo et al., 2023a). This study confirmed that rice varieties can contribute to high yield and efficiency (Meng et al., 2022). The study found that Fyou 498 had a significantly higher population photosynthetic potential, population growth rate, and root vigor than Yixiangyou 2115 during the main growth stages (**Figures 2**, **3**; **Table 4**). This study found that rice varieties and slow-mixed fertilizer base application with urea-N topdressing had significant effects on the photosynthetic characteristics, dry matter accumulation, and transport, and root vigor of mechanical direct-seeding rice. When applying controlled-release fertilizer, it is important to consider the combination of improved varieties and cultivation methods. This study was conducted based on a previous study that found the optimal amount of topdressing N fertilizer for direct-seeding rice in the latter stage to be 20% of the total N application. Increasing the proportion of postponed N fertilizer beyond 20% to 40–60% of the total amount significantly reduces the population quality of direct-seeding rice and increases the lodging index, resulting in yield reduction (Wu et al., 2020; Sun et al., 2022). Therefore, this study did not consider increasing the amount of N fertilizer postponement.

### 4.2 Effect of slow-mixed fertilizer base application combined with available N fertilizer on NUE and rice quality

High-NUE rice varieties, types of N fertilizer, and N fertilizer management practices are closely related to improving both NUE and rice quality (Li et al., 2023; Sun et al., 2023b). In this study, different varieties and slow-mixed basal fertilizer application with urea-N fertilizer management were compared. It was found that Fyou 498 significantly increased the transport of N and the contribution of stem sheaths (leaves) from heading to maturity compared to Yixiangyou 2115. Furthermore, Fyou 498 showed higher NAE and NPE values (**Table 5**), indicating synergistic characteristics of high yield and NUE (Koutroubas and Ntanos, 2003; Sun et al., 2017). However, the head rice rate and taste value of Fyou 498 were significantly lower than those of Yixiangyou 2115, and the chalky kernel rate significantly increased. This study found that the genetic difference in rice quality among varieties was significantly higher than N fertilizer (**Table 6**). This suggests that although the varieties were high yielding and highly efficient, they were not necessarily of high quality. Therefore, it is necessary to increase the screening of high-quality, high-yield, and high-efficiency varieties suitable for mechanization. This finding complements the previous and our research results (Li et al., 2023; Yuan et al., 2023). Previous studies have concluded that the application of controlled-release N fertilizer and optimal N fertilizer operation can increase the N transport rate in plants, promoting N absorption and utilization in rice (Deng et al., 2021; Lyu et al., 2021a; Cheng et al., 2022). However, this study demonstrates that the N transport rate in stem sheaths (leaves) decreases to varying degrees with the delay of the N-topdressing period under the slow-mixed fertilizer base application. This finding is in contrast with the results of previous studies (Deng et al., 2021; Cheng et al., 2022). Although the amount of N transported in the stem sheaths (leaves) and the contribution rate of N transport in the stem sheaths (leaves) increased significantly during the topdressing time with N fertilizer in the experiments, the effect of urea-N fertilizer was significantly improved. However, the proportion of N retained in the stem sheaths (leaves) remained relatively high (**Table 5**). Further research is needed to improve the rate of N transport in vegetative organs during the filling stage of slow-mixed fertilizer combined with urea-N application.

**Table 6:** Effects of slow-mixed fertilizer base application combined with urea topdressing time on rice quality of mechanical direct-seeding hybrid *indica* rice.

Year	Cultivar	N treatments	Brown rice (%)	Milled rice (%)	Head rice (%)	Chalkiness (%)	Chalky kernel (%)	Taste value
2021	Yixiangyou 2115	N_1_	75.00c	64.64c	55.33b	3.11c	12.11c	87.33a
		N_2_	77.41b	66.26b	56.50b	3.32bc	12.63b	84.00c
		N_3_	77.99ab	66.32b	58.26a	3.69ab	14.19a	84.20c
		N_4_	78.06a	67.58a	58.44a	4.00a	14.52a	86.10b
	**Average**		**77.12**	**66.20**	**57.13**	**3.53**	**13.36**	**85.41**
	Fyou 498	N_1_	74.21d	63.11c	48.17c	4.74c	16.48c	82.21a
		N_2_	74.86c	63.78bc	51.02b	6.59b	20.23b	76.33d
		N_3_	76.41b	63.89b	51.91b	7.28a	24.39a	79.00c
		N_4_	77.54a	65.80a	54.61a	7.35a	24.53a	81.05b
	**Average**		**75.76**	**64.15**	**51.57**	**6.49**	**21.41**	**79.65**
	*F* value	V	4.75*	14.87**	52.41**	99.29**	84.20**	72.99**
		N	5.94*	4.71*	8.72*	26.70**	20.52**	9.12**
		V×N	0.53	0.20	1.91	7.88**	6.23**	0.89
2022	Yixiangyou 2115	N_1_	76.29c	63.92c	54.22b	3.47c	12.01b	85.83a
		N_2_	77.71b	65.76b	55.61b	4.14bc	12.97b	81.10c
		N_3_	78.93a	66.42b	57.04a	4.50b	15.51a	83.00b
		N_4_	78.96a	67.67a	58.34a	5.85a	16.87a	85.05a
	**Average**		**77.97**	**65.94**	**56.30**	**4.49**	**14.34**	**83.75**
	Fyou 498	N_1_	70.77d	61.28c	48.69c	8.28c	18.26d	80.71a
		N_2_	72.32c	62.49b	50.95b	8.95c	21.73c	74.50d
		N_3_	74.80b	63.65a	51.62b	10.58b	27.90b	76.67c
		N_4_	75.86a	64.27a	55.03a	11.76a	31.03a	78.69b
	**Average**		**73.44**	**62.92**	**51.43**	**9.64**	**24.73**	**77.64**
	*F* value	V	5.83*	10.14**	47.82**	87.34**	98.04**	54.12**
		N	6.44*	5.22*	6.94*	21.23**	27.11**	10.76**
		V×N	0.24	0.79	3.02	9.01**	7.95**	3.13

N_1_: slow-mixed N fertilizer (120 kg·hm^-2^) as a base; N_2_: N_1_+urea-N (30 kg·hm^-2^) one-time as a base; N_3_: N_1_+urea-N (30 kg·hm^-2^) topdressing at the tillering stage (32d after sowing); N_4_: N_1_+urea-N (30 kg·hm^-2^) topdressing at the booting stage (93d after sowing). V, Variety; N, N fertilizer treatment; V×N, cultivar and N fertilizer treatment interaction. *, *P* < 0.05; **, *P* < 0.01. Different lowercase letters indicate significant (*P* < 0.05) differences among N fertilizer treatments under the same variety.

Research findings on rice quality differences between varieties are more consistent due to their genetic background (Lin et al., 2011). However, the effect of N fertilizer management on rice quality characteristics remains a topic of debate (Cao et al., 2017; Shi et al., 2022; Guo et al., 2023b). Some studies have suggested that using controlled-release N fertilizer, increasing the N application amount, or delaying the application of N fertilizer under the same N application rate can reduce chalkiness and improve the overall eating quality of rice (Yuan et al., 2023; Guo et al., 2023b). Some studies have shown that increasing or delaying the application of N fertilizer can increase the chalkiness of rice (Cao et al., 2017). However, this study found that the effect of combined urea-N on the quality characteristics of direct-seeding rice under slow-mixed fertilizer-based application contradicted previous research (Zhang et al., 2008; Cao et al., 2017; Lyu et al., 2021b). The brown rice, milled rice, head rice, and taste values of direct-seeding rice improved with a delay in the N-topdressing period. The combined application of urea-N under slow-mixed fertilizer-based application may moderately increase grain plumpness (Sun et al., 2017; Sun et al., 2023b). However, delaying the application of urea-N under slow-mixed fertilizer base application worsens the degree of chalkiness and increases the rate of chalky grains. Further research is needed to determine whether the delayed release rate of controlled-release N fertilizer (Cheng et al., 2022) or the moderate delay of urea-N application affects the grain-filling rate (Sun et al., 2023b), starch anabolism (Yuan et al., 2023), and amylopectin chain length distribution (Guo et al., 2023b).

### 4.3 Mechanism of slow-mixed fertilizer base application combined with available N fertilizer synergistic improvement of grain yield, rice quality, and NUE

Previous studies have shown the types and release rates of controlled-release N fertilizers as well as their effectiveness in increasing rice yield through one time basal application (Ke et al., 2018; Wu et al., 2021). These studies also investigated the physiological regulatory mechanisms under different soil types, varieties, and planting methods (Lyu et al., 2021b; Cheng et al., 2022; Meng et al., 2022; Ishfaq et al., 2023). However, there are few studies on the combined application of urea-N fertilizers under slow-mixed fertilizer base applications, especially in mechanical direct-seeding rice. This study aimed to investigate the synergistic effects of urea-N fertilizers on yield, rice quality, and NUE. However, the mechanism underlying this process has rarely been reported. The mechanism for the synergistic improvement of yield, rice quality, and NUE in direct-directing rice by slow-mixed fertilizer basal application with urea-N fertilizer (N_3_ treatment) in this experiment is as follows: different varieties can improve the photosynthetic potential from the jointing to heading stage, optimize the LAI of the population, promote dry matter accumulation and transport during the grain-filling stage, improve the population growth rate from the heading to the maturity stage, and then optimize the yield components, which is an important reason for the final increase in yield. Applying urea-N fertilizer during the booting stage is optimal for slow-mixed fertilizer application. This application can increase the root vigor decay rate from heading to maturity and promote N transport from the stem sheath (leaves) to the panicle during the filling stage. This is the main reason for improving the NUE of urea-N topdressing and enhancing the rice processing and eating quality. It can be used as an important method to achieve high NUE and high-quality coordination in mechanical direct-seeding hybrid *indica* rice.

Selecting appropriate indicators to evaluate grain yield, rice quality, and NUE is crucial for evaluating the physiological ecology of high-yield, high-quality, and NUE crops (Zhang et al., 2013; Sun et al., 2017; Sun et al., 2023b). The use of principal components and correlation analysis is shown in **Figures 4**, **5**. This study suggests that increasing the population’s photosynthetic potential from the jointing to heading stage, promoting N transport in leaves from the heading to maturity stage, and increasing the N transport contribution rate in stem sheaths from the heading to maturity stage, can be used as an evaluation index for the simultaneous improvement of high yield, high quality, and NUE in direct-seeding rice. These findings provide another important way to achieve coordination and unification of high yield, high quality, and NUE in direct-seeding rice.

## 5 Conclusions

The effects of different varieties and slow-mixed fertilizer basal application with urea-N topdressing on the photosynthetic characteristics, dry matter accumulation, and transport, root vigor, NUE, yield, and rice quality of mechanical direct-seeded rice were significant. In this experiment, under the N application level of 150 kg hm^-2^, the combination of slow-mixed fertilizer (N 120 kg hm^-2^) basal application and booting stage urea-N fertilizer (N 30 kg hm^-2^) topdressing significantly improved the photosynthetic potential of different varieties from the jointing to heading stage, the population growth rate and the N transport amount of leaves from the heading to maturity stage. It especially improved the N transport contribution rate in the stem sheaths, achieving the effect of increasing yield. Simultaneously, it improved the NUE of the N fertilizer topdressing and the processing and eating quality of rice synergistically. The best slow-mixed fertilizer basal application with urea-N fertilizer topdressing achieved a synergistic improvement in grain quality, yield, and NUE of direct-seeding rice.

## Data availability statement

The original contributions presented in the study are included in the article/**Supplementary Material**. Further inquiries can be directed to the corresponding author.

## Author contributions

YJS: Data curation, Funding acquisition, Project administration, Writing – original draft. MX: Data curation, Investigation, Writing – review & editing. ZH: Formal analysis, Investigation, Writing – review & editing. YYS: Formal analysis, Methodology, Software, Writing – review & editing. YD: Validation, Visualization, Writing – review & editing. YL: Investigation, Writing – review & editing. XC: Investigation, Software, Writing – review & editing. YC: Investigation, Methodology, Writing – review & editing. WX: Visualization, Writing – review & editing. XH: Data curation, Investigation, Writing – review & editing. PD: Software, Writing – review & editing, Investigation, Methodology. ML: Writing – review & editing, Formal analysis, Software. ZY: Writing – review & editing, Resources, Validation. ZC: Writing – review & editing, Investigation. JM: Supervision, Writing – review & editing.
