# Supplementary material for: Effects of urea topdressing time on yield, nitrogen utilization, and quality of mechanical direct-seeding hybrid indica rice under slow-mixed fertilizer base application
Source: Front Plant Sci. 2024 May 10;15:1400146. doi: 10.3389/fpls.2024.1400146 (PMC11116791; doi:10.3389/fpls.2024.1400146)
Supplement: Supplementary file 1 [file Image_1.pdf]

Supplementary Material

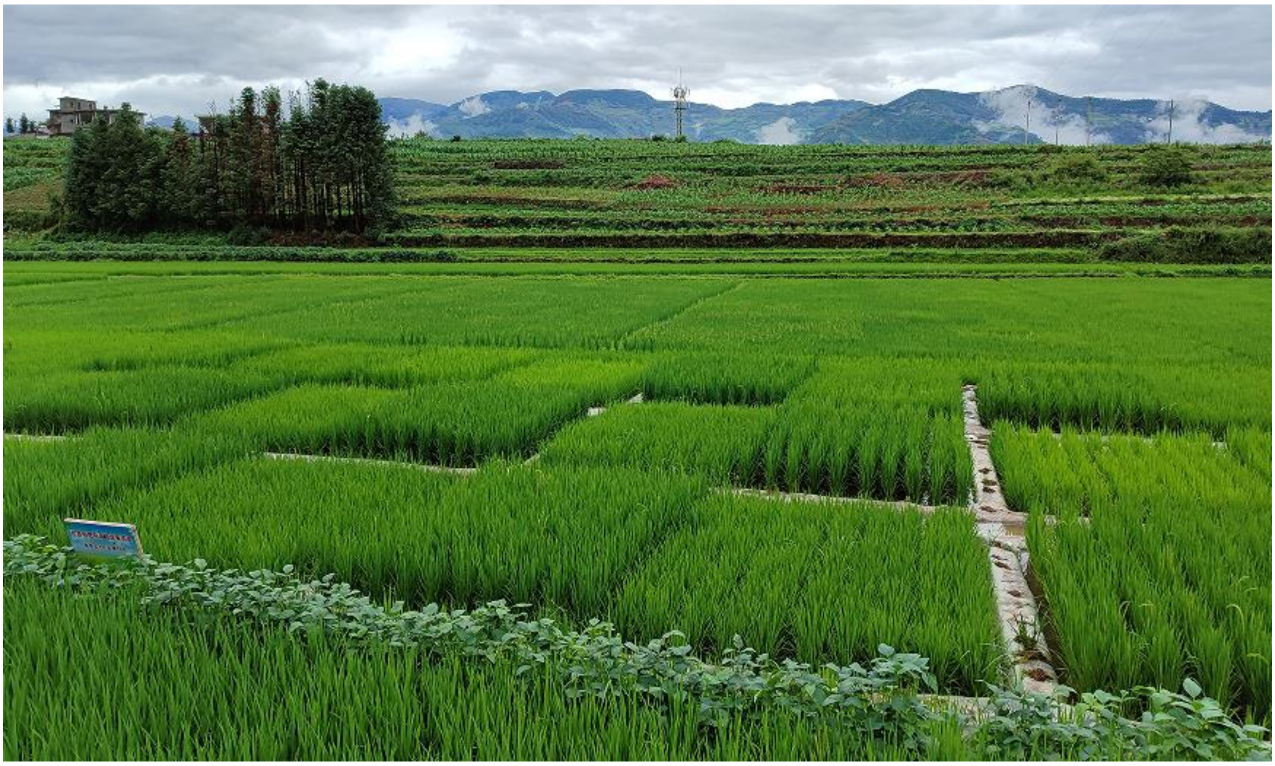

Figure S1. The experiment used a random design of 2 varieties and 4 nitrogen management strategies (field growth at the tillering stage)

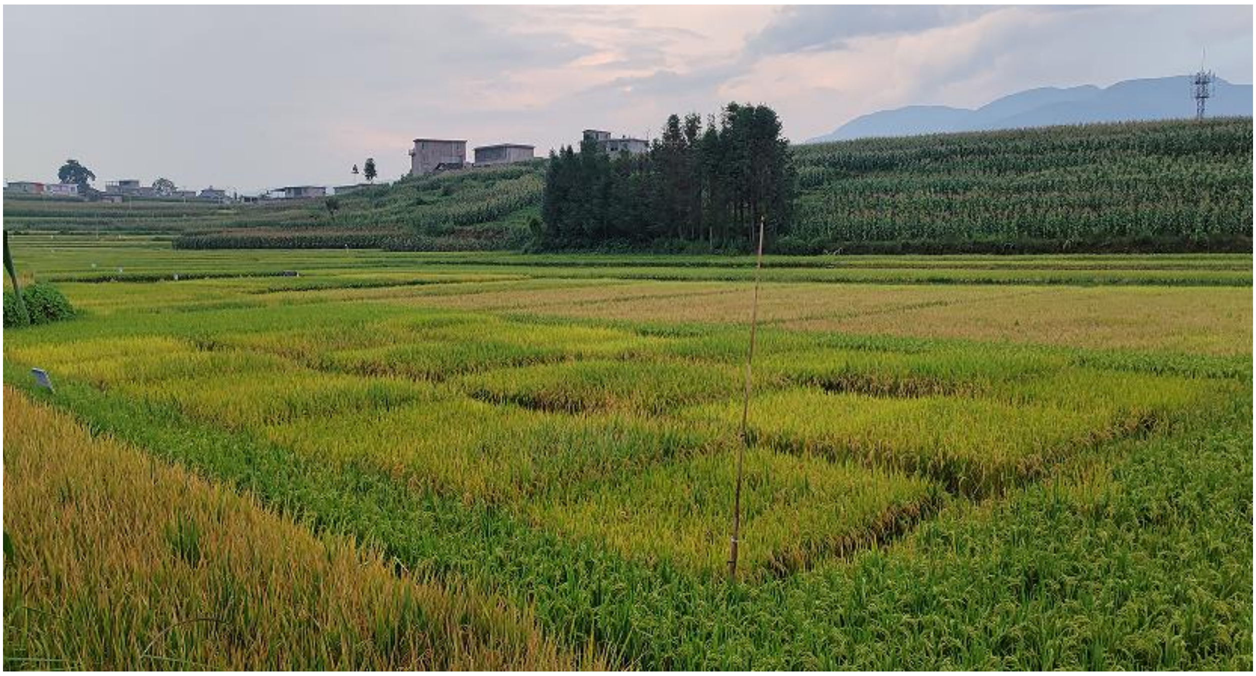

Figure S2. The experiment used a random design of 2 varieties and 4 nitrogen management strategies (field growth at the maturity stage)
