# Supplementary material for: Effects of urea topdressing time on yield, nitrogen utilization, and quality of mechanical direct-seeding hybrid indica rice under slow-mixed fertilizer base application
Source: Front Plant Sci. 2024 May 10;15:1400146. doi: 10.3389/fpls.2024.1400146 (PMC11116791; doi:10.3389/fpls.2024.1400146)
Supplement: Supplementary file 2 [file Table_1.docx]

***Supplementary Material***

**Supplementary TABLE 1** Relative percentage increase or reduce in yield and yield formation of machine- transplanted mechanical direct-seeding hybrid *indica* rice under two cultivars and slow-mixed fertilizer base application combined with urea topdressing time management relative to N_1_ treatment of the same cultivar (%)

| Year | Cultivar | N  treatments | Grain  yield | Effective  panicles | Filled  spikelets | Total  spikelets | Filled  grains |
| --- | --- | --- | --- | --- | --- | --- | --- |
| 2021 | Yixiangyou2115 | N_1_ | 0.00 | 0.00 | 0.00 | 0.00 | 0.00 |
|  |  | N_2_ | 4.42 | 3.99 | 4.76 | 8.66 | -3.04 |
|  |  | N_3_ | 13.20 | 9.93 | 9.86 | 20.82 | 1.00 |
|  |  | N_4_ | 21.29 | 1.91 | 20.07 | 22.39 | 2.97 |
|  | Fyou 498 | N_1_ | 0.00 | 0.00 | 0.00 | 0.00 | 0.00 |
|  |  | N_2_ | 5.41 | 3.95 | 3.29 | 7.32 | -1.05 |
|  |  | N_3_ | 14.43 | 7.64 | 7.68 | 15.83 | 2.11 |
|  |  | N_4_ | 21.60 | 3.46 | 15.23 | 19.17 | 4.05 |
| 2022 | Yixiangyou2115 | N_1_ | 0.00 | 0.00 | 0.00 | 0.00 | 0.00 |
|  |  | N_2_ | 2.93 | 4.92 | 5.77 | 0.80 | -2.55 |
|  |  | N_3_ | 11.80 | 15.54 | 7.06 | 11.88 | 4.78 |
|  |  | N_4_ | 19.39 | 5.00 | 11.80 | 17.89 | 11.31 |
|  | Fyou 498 | N_1_ | 0.00 | 0.00 | 0.00 | 0.00 | 0.00 |
|  |  | N_2_ | 5.03 | 3.94 | 1.32 | 4.94 | -2.17 |
|  |  | N_3_ | 13.76 | 7.65 | 5.56 | 7.46 | 10.31 |
|  |  | N_4_ | 21.67 | 6.17 | 11.69 | 7.83 | 11.32 |

N_1_: slow-mixed N fertilizer (120 kg hm^-2^) as a base; N_2_: N_1_+urea-N (30 kg hm^-2^) one-time as a base; N_3_: N_1_+urea-N (30 kg hm^-2^) topdressing at the tillering stage (32d after sowing); N_4_: N_1_+urea-N (30 kg hm^-2^) topdressing at the booting stage (93d after sowing).

**Supplementary TABLE 2** Relative percentage increase or reduce in LAI at the jointing stage, LAI at the heading stage, photosynthetic potential from the jointing to heading stage, root vigor at the heading stage, root vigor at the maturity stage and root vigor decay rate from the heading to maturity stage of machine-transplanted mechanical direct-seeding hybrid *indica* rice under two cultivars and slow-mixed fertilizer base application combined with urea topdressing time management relative to N_1_ treatment of the same cultivar (%)

| Year | Cultivar | N  treatments | LAI | | Photosynthetic potential from the jointing to heading stage |  | root vigor | | |
| --- | --- | --- | --- | --- | --- | --- | --- | --- | --- |
|  |  |  | Jointing  stage | Heading  stage |  |  | Heading  stage | Maturity  stage | Decay rate from the heading to maturity |
| 2021 | Yixiangyou2115 | N_1_ | 0.00 | 0.00 | 0.00 |  | 0.00 | 0.00 | 0.00 |
|  |  | N_2_ | 23.51 | 22.26 | 15.73 |  | 27.05 | 12.05 | -8.18 |
|  |  | N_3_ | 32.67 | 33.80 | 39.51 |  | 53.36 | 39.08 | -11.31 |
|  |  | N_4_ | 21.31 | 26.98 | 51.14 |  | 80.10 | 77.89 | -16.29 |
|  | Fyou 498 | N_1_ | 0.00 | 0.00 | 0.00 |  | 0.00 | 0.00 | 0.00 |
|  |  | N_2_ | 21.74 | 20.63 | 14.41 |  | 40.43 | 27.61 | -10.77 |
|  |  | N_3_ | 27.62 | 28.26 | 31.36 |  | 76.27 | 65.91 | -15.12 |
|  |  | N_4_ | 21.15 | 25.41 | 43.03 |  | 118.52 | 126.18 | -21.44 |
| 2022 | Yixiangyou2115 | N_1_ | 0.00 | 0.00 | 0.00 |  | 0.00 | 0.00 | 0.00 |
|  |  | N_2_ | 21.38 | 18.19 | 1.57 |  | 24.79 | 43.46 | -11.30 |
|  |  | N_3_ | 28.25 | 29.34 | 34.86 |  | 50.24 | 82.19 | -15.96 |
|  |  | N_4_ | 17.27 | 22.75 | 46.10 |  | 75.10 | 121.55 | -19.90 |
|  | Fyou 498 | N_1_ | 0.00 | 0.00 | 0.00 |  | 0.00 | 0.00 | 0.00 |
|  |  | N_2_ | 15.63 | 16.17 | 18.72 |  | 10.51 | 32.74 | -14.90 |
|  |  | N_3_ | 25.50 | 23.67 | 13.63 |  | 37.16 | 82.91 | -24.65 |
|  |  | N_4_ | 14.32 | 21.36 | 53.48 |  | 72.09 | 133.80 | -30.09 |

N_1_: slow-mixed N fertilizer (120 kg hm^-2^) as a base; N_2_: N_1_+urea-N (30 kg hm^-2^) one-time as a base; N_3_: N_1_+urea-N (30 kg hm^-2^) topdressing at the tillering stage (32d after sowing); N_4_: N_1_+urea-N (30 kg hm^-2^) topdressing at the booting stage (93d after sowing).

**Supplementary TABLE 3** Relative percentage increase or reduce in NUE, rice quality of machine-transplanted mechanical direct-seeding hybrid *indica* rice under two cultivars and slow-mixed fertilizer base application combined with urea topdressing time management relative to N_4_ treatment of the same cultivar (%)

| Year | Cultivar | N  treatments | NUE | |  | Rice quality | | | |
| --- | --- | --- | --- | --- | --- | --- | --- | --- | --- |
|  |  |  | NAE | NRE |  | Head rice | Chalkiness | Chalky kernel | Taste value |
| 2021 | Yixiangyou2115 | N_1_ | - | - |  | -3.11 | -0.89 | -2.41 | 1.43 |
|  |  | N_2_ | -45.56 | -10.52 |  | -1.94 | -0.68 | -1.89 | -2.44 |
|  |  | N_3_ | -21.86 | -3.91 |  | -0.18 | -0.31 | -0.33 | -2.21 |
|  |  | N_4_ | 0.00 | 0.00 |  | 0.00 | 0.00 | 0.00 | 0.00 |
|  | Fyou 498 | N_1_ | - | - |  | -6.44 | -2.61 | -8.05 | 1.43 |
|  |  | N_2_ | -44.80 | -7.40 |  | -3.59 | -0.76 | -4.30 | -5.82 |
|  |  | N_3_ | -19.85 | -2.13 |  | -2.70 | -0.07 | -0.14 | -2.53 |
|  |  | N_4_ | 0.00 | 0.00 |  | 0.00 | 0.00 | 0.00 | 0.00 |
| 2022 | Yixiangyou2115 | N_1_ | - | - |  | -4.12 | -2.38 | -4.86 | 0.92 |
|  |  | N_2_ | -43.91 | -11.88 |  | -2.73 | -1.71 | -3.90 | -4.64 |
|  |  | N_3_ | -20.24 | -3.78 |  | -1.30 | -1.35 | -1.36 | -2.41 |
|  |  | N_4_ | 0.00 | 0.00 |  | 0.00 | 0.00 | 0.00 | 0.00 |
|  | Fyou 498 | N_1_ | - | - |  | -6.34 | -3.48 | -12.77 | 2.57 |
|  |  | N_2_ | -44.79 | -8.16 |  | -4.08 | -2.81 | -9.30 | -5.32 |
|  |  | N_3_ | -21.28 | -2.96 |  | -3.41 | -1.18 | -3.13 | -2.57 |
|  |  | N_4_ | 0.00 | 0.00 |  | 0.00 | 0.00 | 0.00 | 0.00 |

N_1_: slow-mixed N fertilizer (120 kg hm^-2^) as a base; N_2_: N_1_+urea-N (30 kg hm^-2^) one-time as a base; N_3_: N_1_+urea-N (30 kg hm^-2^) topdressing at the tillering stage (32d after sowing); N_4_: N_1_+urea-N (30 kg hm^-2^) topdressing at the booting stage (93d after sowing).
